# Additive or Synergistic Interactions Between IL-17A or IL-17F and TNF or IL-1β Depend on the Cell Type

**DOI:** 10.3389/fimmu.2019.01726

**Published:** 2019-07-23

**Authors:** Mélissa Noack, Audrey Beringer, Pierre Miossec

**Affiliations:** Immunogenomics and Inflammation Research Unit, EA 4130, Edouard Herriot Hospital, Hospices Civils de Lyon and University Claude Bernard Lyon 1, Lyon, France

**Keywords:** IL-17A, IL-17F, synergistic effect, additive effect, cytokine production, different cell types

## Abstract

**Background:** IL-17A has effects on several cell types and is a therapeutic target in several inflammatory diseases. IL-17F shares 50% homology and biological activities with IL-17A. It is now of interest to target both cytokines. The objective was to compare the IL-17A and IL-17F effect on cytokine production by RA synoviocytes, and to extend to other cells.

**Methods:** Cells (RA synoviocytes, psoriasis skin fibroblasts, endothelial cells, myoblasts, and hepatocytes) were cultured in the presence or not of: IL-17A, IL-17F, TNF, IL-1β alone or their combinations, IL-17A/TNF, IL-17A/IL-1β, IL-17A/TNF/IL-1β, IL-17F/TNF, IL-17F/IL-1β, and IL-17F/TNF/IL-1β. All experiments were performed in parallel to reduce variability. After 48 h, supernatants were recovered and IL-6 and IL-8 levels were measured by ELISA.

**Results:** IL-17A and IL-17F alone increased significantly IL-6 and IL-8 productions by synoviocytes, with a stronger effect for IL-17A. For IL-6 production, TNF or IL-1β alone had the largest effect on myoblasts (5-fold increase), while for IL-8 production, it was on skin fibroblasts (5-fold increase). The IL-17A/TNF synergistic increase was observed on all cells for IL-6; and for IL-8, except for endothelial cells. For IL-17F/TNF, except with endothelial cells, a synergistic effect was also observed, but less powerful than with IL-17A/TNF. IL-17A/IL-1β or IL-17F/IL-1β effect was cell-type dependent, with an additive effect for synoviocytes (1.6 and 2-fold increase, respectively for IL-6, and 1.8 and 2-fold increase, respectively for IL-8) and a synergistic effect for hepatocytes (3.8 and 4.2-fold increase, respectively for IL-6, and 6 and 2-fold increase, respectively for IL-8). The three-cytokine combination induced an additive effect for synoviocytes and a synergistic effect for skin fibroblasts.

**Conclusion:** IL-17A and IL-17F acted similarly by inducing pro-inflammatory cytokine secretion, with a stronger response intensity with IL-17A. Their activities were potentiated by the combination with TNF and IL-1β, with an effect dependent on the cell type.

## Introduction

IL-17A is a critical cytokine in the immune response against extra-cellular bacteria and fungi. However, an excess of IL-17A is associated with many inflammatory disorders. IL-17A is now a registered target in psoriasis, psoriatic arthritis, ankylosing spondylitis (1). Furthermore, the IL-17 family has been expanding with focus specifically on IL-17F. IL-17F is the closest member to IL-17A, with a 50% homology and rather similar biological functions. Even if IL-17A has a stronger effect than IL-17F, both cytokines induce common transducing pathways through a common receptor composed by two chains, IL-17RA and IL-17RC (1). IL-17F could also be an interesting therapeutic target, even more associated to IL-17A inhibition with double specific inhibitors (2).

IL-17A was first linked to inflammation through the induction of IL-6 production by fibroblasts (3) and synoviocytes (4, 5). The major role of IL-17A is the production of pro-inflammatory mediators by many types of cells, such as macrophages, dendritic cells, fibroblasts, osteoblasts, synoviocytes, chondrocytes, endothelial, or epithelial cells (6). During rheumatoid arthritis (RA), IL-17A is spontaneously produced by the synovium (4) and this increased IL-17A level correlates with disease activity (7). Both IL-17A and IL-17F have been found to be expressed in RA synovial tissue with a stronger expression for IL-17F (8). IL-17A alone had a limited effect, but can synergize with other pro-inflammatory cytokines such as IL-1 or TNF that leads to increased inflammation (8–10). These synergistic effects have been demonstrated in many cell types like synoviocytes or myoblasts (11). Using microarray studies, a synergistic effect between IL-17F and TNF was also demonstrated, reaching a level of pro-inflammatory gene signature not very far from that induced by the synergistic effect of TNF and IL-17A (8, 12).

The effects of IL-17A and IL-17F and their interactions with other cytokines have been mainly shown in synoviocytes but also in other cell types, but in independent studies. Thus, our objective was to compare in parallel the effects of the same batch of IL-17A and IL-17F, in combination or not with TNF and IL-1β, on pro-inflammatory cytokine production (IL-6 and IL-8) by several cell types [RA synoviocytes, psoriatic skin fibroblasts, myoblasts, hepatocytes and human umbilical vein endothelial cells (HUVEC)], with the effect on synoviocytes as reference. The focus was on differences between cytokine interactions related to the cell type.

## Materials and Methods

### Samples

RA synoviocytes were obtained from synovial tissue of RA patients undergoing joint surgery and who fulfilled the American College of Rheumatology criteria for RA (13). Synovial tissue was minced into small pieces and then adhered in 6-well plates in Dulbecco's modified Eagle's medium (DMEM; Eurobio, Courtaboeuf, France) supplemented with 10% fetal bovine serum (FBS; Life Technologies, Carlsbad, USA), 2 mM L-glutamine and 100 U/ml penicillin/streptomycin (Eurobio). Cells were maintained at 37°C in a humidified 5% carbon dioxide incubator and used between passages 4–9.

Skin fibroblasts were obtained from skin biopsies of psoriatic patients who fulfilled the Classification Criteria for Psoriasis or Psoriatic Arthritis (CASPAR). Biopsies from lesional or non lesional skin were minced into small pieces and adhered in 6-well plates in Dulbecco's modified Eagle's medium (DMEM; Eurobio, Courtaboeuf, France) supplemented with 10% fetal bovine serum (FBS; Life Technologies, Carlsbad, USA), 2 mM L-glutamine and 100 U/ml penicillin/streptomycin (Eurobio). Cells were maintained at 37°C in a humidified 5% CO_2_ incubator and used between passages 4 to 9.

Muscle samples were obtained from subjects undergoing orthopedic surgery. Biopsies were performed on m. vastus lateralis (femoral quadriceps) at distance of the joint. After surgery, muscle samples were immediately placed in sterile PBS with antibiotics (penicillin and streptomycin, Eurobio, Courtaboeuf, France) and washed. The fat and fibrous tissues were removed. Muscle samples were cut into fragments (1–2 mm^3^) and incubated at 37°C for 30 min with 1 mg/mL collagenase (Sigma-Aldrich, St. Louis, MO, USA). After washing and filtration, a first selection was done to remove fibroblasts by incubating the supernatants in petri dishes at 37°C for 1 h. Unattached myoblasts were then transferred and cultured at 37°C/5% CO_2_ in Ham's-F10 medium (Eurobio) supplemented with 20% fetal bovine serum (Life Technologies, Carlsbad, USA), 2% Penicillin-Streptomycin (Eurobio), 1% L-glutamine (Eurobio) and 1% Amphotericin B (Eurobio). After 10 days, adherent cells were detached with trypsin (Eurobio), and myoblasts were purified by positive selection with CD56 microbeads (Miltenyi Biotech, Bergisch Gladbach, Germany), according to the instructions of the manufacturer. Myoblasts were used between passages 2 and 8.

Human umbilical vein endothelial cells (HUVEC) were collected from umbilical cords by collagenase perfusion of umbilical veins. The cells were maintained at 37 C/5% CO_2_ in endothelial cell basal medium (EBM-2; Lonza, Cologne, Germany) enriched with endothelial growth medium (EGM-2) bullet kit (Lonza, Cologne, Germany), 2% penicillin/streptomycin (Eurobio, Courtaboeuf, France) and 1% Amphotericin B (Eurobio).

For the different cell types, each individual signed an informed consent form. The protocol was approved by the Ethics Committee of the Hospitals of Lyon for the protection of persons participating in biomedical research under number AC-2016-2729.

### Cell Culture Conditions

The human hepatoma HepaRG cells (cell line) were seeded at a density of 20,000 cells/cm^2^ and used after 15 days post-plating. HepaRG cells were grown in William's E medium (Sigma, St. Louis, MO, USA) supplemented with 10% fetal bovine serum (Life Technologies), 2 mM L-glutamine (Eurobio), 5 μg/mL insulin (Sigma), 50 μM hydrocortisone hemisuccinate (Serb, Paris, France), 50 U/mL penicillin and 50 μg/mL streptomycin (Eurobio).

Synoviocytes, skin fibroblasts, myoblasts and HUVEC were seeded overnight in 96-well plates at a density of 2 × 10^4^ cells/well.

Cells were treated with 50 ng/ml of IL-17A or IL-17F (UCB Pharma, Berkshire, United Kingdom), 1 ng/ml of TNFα (R&D Systems, Minneapolis, USA) or 10 pg/ml of IL-1β (R&D Systems, Minneapolis, USA) alone or in combination. Supernatants were collected after 48 h of culture and cytokine level was measured.

### Enzyme-Linked Immunosorbent Assays (ELISAs)

IL-6 and IL-8 productions were evaluated from culture supernatants with commercially available Duoset ELISA kits, according to the manufacturer's instructions (R&D system, Minneapolis, USA). The detectable range of IL-6 was from 600 to 9.38 pg/ml and for IL-8 it was from 2,000 to 31.3 pg/ml. The dilution factor for the samples was: synoviocytes and skin fibroblasts: 1/1,000 for IL-6 and IL-8; endothelial cells: 1/25 for IL-6 and 1/100 for IL-8; myoblasts: 1/500 for IL-6 and 1/250 for IL-8; hepatocytes: 1/10 for IL-6 and 1/100 for IL-8.

### Statistical Analysis

An additive effect is defined if the value of the cytokine combination is equal or lower than the sum of the values with each cytokine alone. A synergistic effect is defined if the value of the combination is higher than the sum of the values with each cytokine alone.

Statistical analyses were performed using paired Wilcoxon test. All analyses were performed with Graph Pad Prism 6 software. *p*-values ≤0.05 were considered as significant.

## Results

### Effects on RA Synoviocytes

IL-17A and IL-17F are known to induce IL-6 production by synoviocytes in a dose-dependent manner (8). To set the system, synoviocytes were first used to compare the effects of both cytokines at 50 ng/ml on IL-6 and IL-8 production by RA synoviocytes.

**Figure 1:**
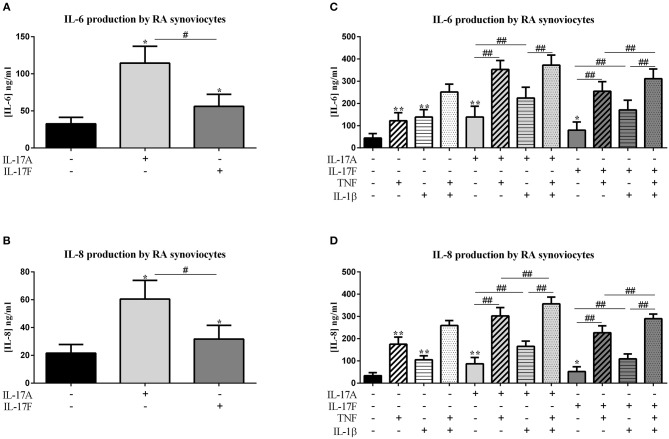
Effect of cytokine interactions on synoviocytes. Synoviocytes were cultured during 48 h in presence or not of different cytokine treatments: IL-17A (50 ng/ml), IL-17F (50 ng/ml), TNF (1 ng/ml), or IL-1β (10 pg/ml), cytokine alone or in combination, IL-17A+TNF, IL-17F+TNF, IL-17A+IL-1β, IL-17F+IL-1β, IL-17A+TNF+IL-1β, or IL-17F+TNF+IL-1β. The production of IL-6 and IL-8 was measured by ELISA in the supernatants after 48 h. ^*^p ≤ 0.05, ^*^compares with control, ^#^compares conditions. Results are represented as mean ± SEM, **(A,B)** *n* = 7, **(C,D)**, *n* = 3. Triplicates were done in each experiment. ^**^,^*##*^*p* ≤ 0.01.

IL-17A and IL-17F increased significantly the production of IL-6 and IL-8 compared to control (IL-17A: IL-6, *p* ≤ 0.0001 and IL-8, *p* ≤ 0.0001; IL-17F: IL-6, *p* = 0.0001 and IL-8, *p* = 0.0004; Figures 1A,B). As expected, the cytokine production was increased even stronger with IL-17A than with IL-17F (IL-6, *p* ≤ 0.004 and IL-8, *p* ≤ 0.004).

In addition, IL-17A can synergize with other cytokines such as TNF or IL-1β (8, 9) and IL-17F also (8). Thus, the impact of the combination between IL-17A or IL-17F, and TNF or IL-1β, or the three cytokines was evaluated. Previously defined concentrations of these cytokines were used, 50 ng/ml for IL-17A and IL-17F, 1 ng/ml for TNF, and 10 pg/ml for IL-1β (10, 14). Cytokines alone increased IL-6 production by synoviocytes (*p* ≤ 0.004, Figure 1C). The effect of TNF, IL-1β and IL-17A was similar, and IL-17F was 40% less active (TNF: 122.5 ± 36.1 ng/ml; IL-1β: 138.6 ± 33.9 ng/ml; IL-17A: 138.8 ± 48.7 ng/ml; IL-17F: 79.6 ± 37.0 ng/ml for IL-17F alone).

The combination of TNF and IL-1β induced an additive effect on IL-6 release (*p* ≤ 0.004, Figure 1C). This additive effect was also observed between IL-17A and IL-1β (*p* ≤ 0.004, Figure 1C) while there was a synergistic effect between IL-17A and TNF (*p* ≤ 0.004, Figure 1C). The combination of the three cytokines induced an IL-6 production slightly higher than IL-17A plus TNF but significantly higher compared to IL-1β plus IL-17A (*p* ≤ 0.004, Figure 1C).

For IL-17F, a synergistic effect was observed between IL-17F and TNF (*p* ≤ 0.004, Figure 1C) and an additive effect with IL-1β (*p* ≤ 0.004, Figure 1C). The combination of the three cytokines induced a higher IL-6 production than both combinations of two cytokines (IL-17F/TNF or IL-17F/IL-1β; *p* ≤ 0.008, Figure 1C).

IL-8 production was induced by the four cytokines alone, with the highest effect with TNF (*p* ≤ 0.04, Figure 1D). As for IL-6 production, the combination of TNF and IL-1β induced an additive effect on IL-8 secretion (*p* ≤ 0.008, Figure 1D), likewise the combination of IL-17A and IL-1β (*p* ≤ 0.004, Figure 1D) and the combination of IL-17F and TNF (*p* ≤ 0.004, Figure 1D). Otherwise, a synergistic effect was observed between IL-17A and TNF (*p* ≤ 0.004, Figure 1D). The combination of IL-1β and IL-17F induced the same effect as IL-1β alone. The combination of three cytokines, IL-17A/TNF/IL-1β or IL-17F/TNF/IL-1β induced an increase of IL-8 production compared to the combination of two cytokines (IL-17A/TNF or IL-17A/IL-1β and IL-17F/TNF or IL-17F/IL-1β, respectively, *p* ≤ 0.008, Figure 1D).

In conclusion, on synoviocytes, the four cytokines used alone induced pro-inflammatory cytokine secretion, with the lowest effect for IL-17F. The combination of IL-17A and TNF had a synergistic effect on both IL-6 and IL-8 production, while the combination of IL-17A and IL-1β had an additive effect. A synergistic effect was also observed between IL-17F and TNF on IL-6 secretion while it was only an additive effect on IL-8 release. The combination IL-1β and IL-17F induced an additive effect on IL-6 and no additional effect on IL-8 than IL-1β alone.

### Effects on Skin Fibroblasts

**Figure 2:**
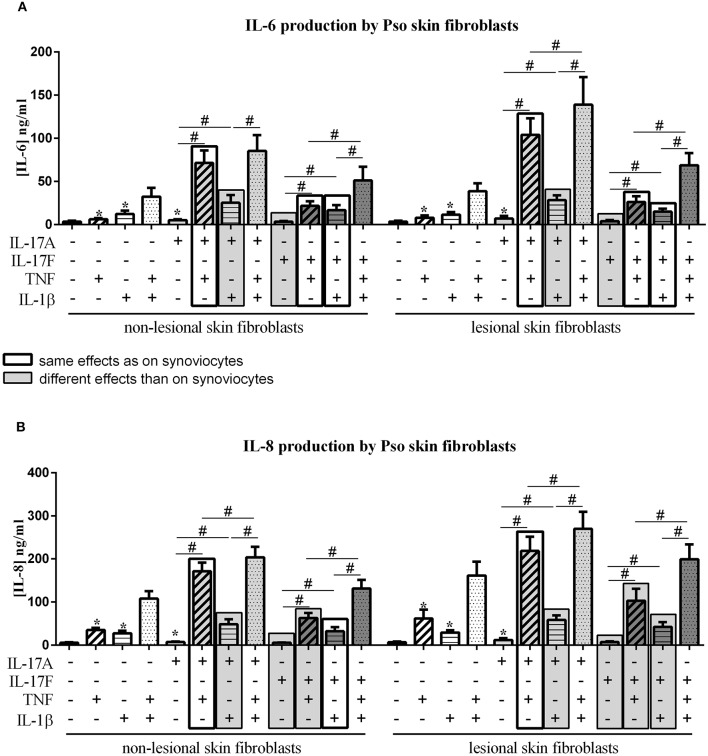
Effect of cytokine interactions on skin fibroblasts. Skin fibroblasts were cultured during 48 h in presence or not of different cytokine treatments: IL-17A (50 ng/ml), IL-17F (50 ng/ml), TNF (1 ng/ml) or IL-1β (10 pg/ml), cytokine alone or in combination, IL-17A+TNF, IL-17F+TNF, IL-17A+IL-1β, IL-17F+IL-1β, IL-17A+TNF+IL-1β or IL-17F+TNF+IL-1β. The production of IL-6 **(A)** and IL-8 **(B)** was measured by ELISA in the supernatants after 48 h. ^*^p ≤ 0.05, ^*^compares with control, ^#^compares conditions. Results are represented as mean ± SEM. *n* = 3. Triplicates were done in each experiment.

Psoriasis is another disease with a major role of IL-17A. The aim was to compare the effect of IL-17A and IL-17F, in combination or not with TNF and IL-1β on skin fibroblasts vs. synoviocytes. Furthermore, fibroblasts from the same patient but from two biopsies, one from non-lesional skin and one from lesional skin were compared. The induced effects were compared to those induced on synoviocytes, same effects were represented by black frame and different effects were represented by filled gray frame in Figure 2.

IL-17A, TNF and IL-1β alone induced a significant increase of IL-6 and IL-8 production, in both non-lesional skin fibroblasts (NLSF) and lesional skin fibroblasts (LSF) compared to control (*p* ≤ 0.03, Figures 2A,B). This induction was stronger for IL-8 compared to IL-6. IL-17F alone had no effect on IL-6 and IL-8 secretion. The combination of TNF and IL-1β had a synergistic effect on IL-6 and IL-8 release in NLSF and in LSF, differently from synoviocytes in which TNF and IL-1β induced an additive effect.

Furthermore, with both IL-17A and IL-17F, a synergistic effect with TNF was observed in NLSF for IL-6 (*p* ≤ 0.02, Figure 2A) and for IL-8 (*p* ≤ 0.02, Figure 2B) and in LSF for IL-6 (*p* ≤ 0.02, Figure 2A) and for IL-8 (*p* ≤ 0.02, Figure 2B). This was slightly different from synoviocytes in which a synergistic effect was observed between IL-17A and TNF for IL-6 and IL-8, while the combination IL-17F/TNF induced a synergistic effect for IL-6 but only an additive effect on IL-8 secretion.

Unlike synoviocytes, the combination of IL-17A and IL-1β induced a synergistic effect in both NLSF and LSF on IL-6 and IL-8 release (*p* ≤ 0.04, Figures 2A,B).

As for synoviocytes, the combination of IL-17F and IL-1β induced only an additive effect, but not a synergistic effect as for IL-17A. This additive effect was observed in NLSF and in LSF on the production of IL-6 and IL-8 (NLSF: IL-6, *p* ≤ 0.03, Figure 2A; IL-8, *p* ≤ 0.02, Figure 2B; LSF: IL-6, *p* ≤ 0.03, Figure 2A; IL-8, *p* ≤ 0.02, Figure 2B).

Similarly to synoviocytes, the combination of the three cytokines, IL-17A/TNF/IL-1β or IL-17F/TNF/IL-1β induced a higher production of both IL-6 and IL-8 in NLSF and in LSF, compared to cytokine alone or in combination two by two (*p* ≤ 0.05, Figures 2A,B), except for IL-6 with IL-17A/TNF vs. IL-17A/TNF/IL-1β in NLSF where the increase with the three cytokines was not significant (*p* > 0.05).

In conclusion with skin fibroblasts, IL-17A alone but not IL-17F induced a significant increase of IL-6 and IL-8 production, while both cytokines induced IL-6 and IL-8 secretion in synoviocytes. IL-17A acted in synergy with both TNF and IL-1β, unlike synoviocytes where IL-17A acted in synergy only with TNF. IL-17F acted in synergy only with TNF on IL-6 and IL-8 production while it was only on IL-6 secretion for synoviocytes; and in an additive way with IL-1β on IL-6 and IL-8 release, similarly to synoviocytes. Furthermore, the cytokine effects on NLSF and LSF were similar, but a higher pro-inflammatory production by LSF than by NLSF was observed, mainly with TNF, which alone induced a 2-fold higher release of IL-8 by LSF.

### Effects on HUVEC

**Figure 3:**
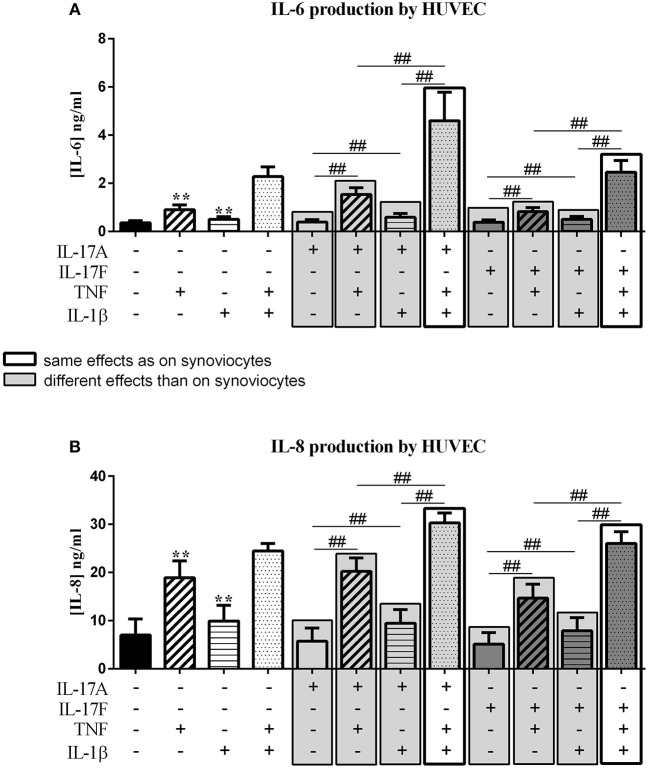
Effect of cytokine interactions on endothelial cells. Endothelial cells (HUVEC) were cultured during 48 h in presence or not of different cytokine treatments: IL-17A (50 ng/ml), IL-17F (50 ng/ml), TNF (1 ng/ml) or IL-1β (10 pg/ml), cytokine alone or in combination, IL-17A+TNF, IL-17F+TNF, IL-17A+IL-1β, IL-17F+IL-1β, IL-17A+TNF+IL-1β or IL-17F+TNF+IL-1β. The production of IL-6 **(A)** and IL-8 **(B)** was measured by ELISA in the supernatants after 48 h. ^*^p ≤ 0.05, ^*^compares with control, ^#^compares conditions. Results are represented as mean ± SEM. *n* = 3. Triplicates were done in each experiment. ^**^,^*##*^*p* ≤ 0.01.

During any chronic inflammatory disorder, cardiovascular events are more common and IL-17 is linked to this increased cardiovascular risk (15, 16). IL-17 acts on different cell types of the cardiovascular system (15) and notably it induces pro-inflammatory cytokine production from endothelial cells (5). In this context, the effects of IL-17A and IL-17F were compared, alone and in combination with TNF or IL-1β, to those on synoviocytes (same effects were represented by black frame and different effects were represented by filled gray frame in Figure 3)

Unlike synoviocytes, TNF and IL-1β alone but not IL-17A and IL-17F induced a significant increase of IL-6 and IL-8 secretion (*p* ≤ 0.004, Figure 3). Unlike synoviocytes, the combination TNF/IL-1β had a synergistic effect on IL-6 secretion (*p* ≤ 0.02, Figure 3A), while for IL-8 production, as for synoviocytes, TNF/IL-1β had only an additive effect (*p* ≤ 0.02, Figure 3B).

IL-17A in combination with TNF increased IL-6 release (*p* ≤ 0.004, Figure 3A) but the intensity was lower than induced in synoviocytes. IL-6 production with IL-17A/IL-β stimulation was higher than IL-17A stimulation alone (*p* ≤ 0.004, Figure 3A).

Unlike IL-17A, the combination with IL-17F, IL-17F/TNF, or IL-17F/IL-1β induced the same IL-6 level as TNF and IL-1β alone, respectively (*p* > 0.05, Figure 3A). The combination of IL-17A and TNF or IL-1β had a moderate effect on IL-6 secretion while the combination of the three cytokines, IL-17A/TNF/IL-1β or IL-17F/TNF/IL-1β, induced a synergistic effect on IL-6 level, with a stronger effect with IL-17A compared to IL-17F (*p* ≤ 0.004, Figure 3A).

For IL-8, the combination of the three cytokines had an additive effect. The combination of TNF/IL-1β, as for synoviocytes, had also an additive effect on IL-8 production (*p* ≤ 0.05, Figure 3B). The combination of IL-17A with TNF or IL-1β, had a similar effect on IL-8 level as TNF or IL-1β alone, respectively (*p* > 0.05, Figure 3B), unlike in synoviocytes where IL-17A/TNF induced a synergistic effect and IL-7A/IL-1β an additive effect on IL-8. As for IL-17A, the combinations IL-17F/TNF and IL-17F/IL-1β had the same effect as TNF and IL-1β alone, respectively (*p* > 0.05, Figure 3B).

In conclusion for HUVEC, unlike synoviocytes, TNF and IL-1β alone but not IL-17A and IL-17F induced IL-6 and IL-8 secretion. As for synoviocytes, the combination of IL-17A/TNF/IL-1β or IL-17F/TNF/IL-1β had the major effect on IL-6 production. For IL-8 secretion, unlike synoviocytes, IL-17A or IL-17F alone had no significant effect on cytokine production, but increased the effects of TNF and IL-1β.

### Effects on Myoblasts

**Figure 4:**
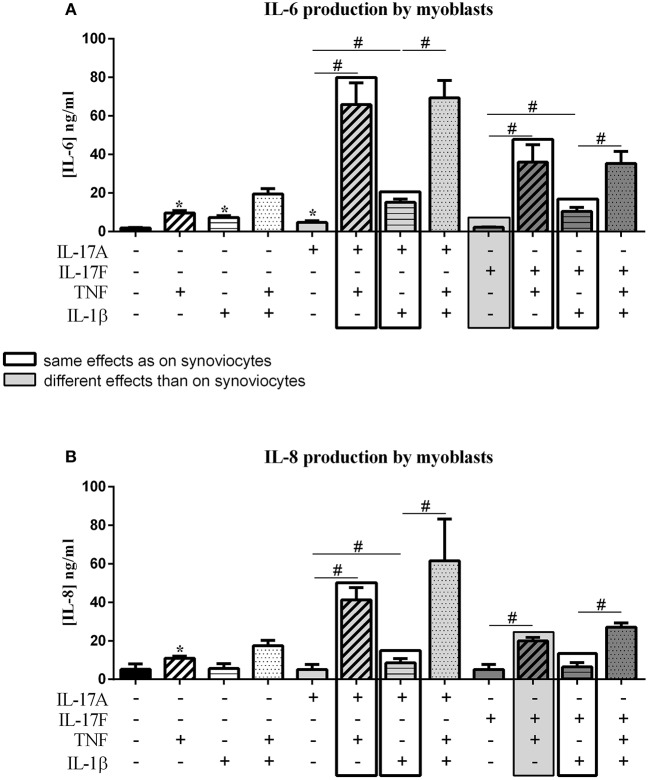
Effect of cytokine interactions on myoblasts. Myoblasts were cultured during 48 h in presence or not of different cytokine treatments: IL-17A (50 ng/ml), IL-17F (50 ng/ml), TNF (1 ng/ml) or IL-1β (10 pg/ml), cytokine alone or in combination, IL-17A+TNF, IL-17F+TNF, IL-17A+IL-1β, IL-17F+IL-1β, IL-17A+TNF+IL-1β or IL-17F+TNF+IL-1β. The production of IL-6 **(A)** and IL-8 **(B)** was measured by ELISA in the supernatants after 48 h. ^*^p ≤ 0.05, ^*^compares with control, ^#^compares conditions. Results are represented as mean ± SEM. *n* = 3. Duplicates were done in each experiment.

Immune infiltrates with Th17 cells are seen in myositis and a previous paper from the laboratory showed that IL-17 acts on myoblasts to increase the effects of IL-1β (17). The aim was to test the effect of IL-17A vs. IL-17F and of the combination with TNF and IL-1β on myoblasts. The induced effects were compared to those induced on synoviocytes, same effects were represented by black frame and different effects were represented by filled gray frame in Figure 4.

As for synoviocytes, TNF alone increased the production of both IL-6 and IL-8 while IL-17A and IL-1β alone had a significant effect only on IL-6 secretion (IL-6, *p* ≤ 0.03, Figure 4A; IL-8, *p* ≤ 0.03, Figure 4B). Unlike synoviocytes, IL-17F alone had no effect.

As for synoviocytes, the combination of IL-1β and TNF induced an additive effect on IL-6 and IL-8 production. The combination of IL-17A and IL-1β had only an additive effect (IL-6, *p* ≤ 0.03, Figure 4A; IL-8, *p* ≤ 0.03, Figure 4B). As for synoviocytes, the combination IL-17A and TNF had a synergistic effect on both IL-6 and IL-8 (IL-6, *p* ≤ 0.03, Figure 4A; IL-8, *p* ≤ 0.03, Figure 4B).

As for IL-17A, the combination of IL-17F and TNF but not IL-1β induced a synergistic effect (IL-6, *p* ≤ 0.03, Figure 4A; IL-8, *p* ≤ 0.03, Figure 4B). In synoviocytes, IL-17F/TNF combination induced a synergistic effect only on IL-6 but not on IL-8 which was an additive effect, as IL-17F/IL-1β combination.

For both IL-17A and IL-17F, the combination of the three cytokines induced a similar effect as IL-17/TNF combination for IL-6 secretion (*p* > 0.05, Figure 4A) while for synoviocytes an increase was observed.

For IL-8 production, as for synoviocytes, the combination of IL-17A, TNF, and IL-1β induced an increased secretion compared to the combination of cytokines two by two (Figure 4B). The same effect was observed with IL-17F (*p* ≤ 0.05 Figure 4B). Furthermore, the production of IL-6 or IL-8 was higher in condition with IL-17A compared to IL-17F, as for synoviocytes.

In conclusion for myoblasts, as for synoviocytes, IL-17A acted in synergy with TNF and in an additive way with IL-1β on IL-6 and IL-8 release. IL-17F alone had no effect but synergized with TNF to induce IL-6 and IL-8 secretion, while in synoviocytes IL-17F induced both IL-6 and IL-8 cytokine productions and synergized only with TNF to induce IL-6. The combination IL-17F/IL-1β induced an additive effect on IL-6 and IL-8 productions, as for synoviocytes.

### Effects on Hepatocytes

**Figure 5:**
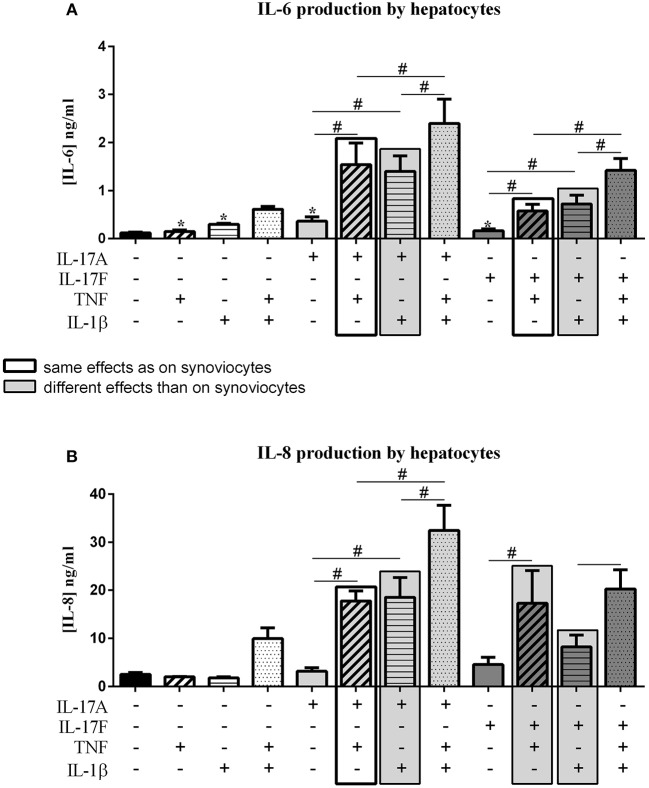
Effect of cytokine interactions on hepatocytes. Hepatocytes were cultured during 48 h in presence or not of different cytokine treatments: IL-17A (50 ng/ml), IL-17F (50 ng/ml), TNF (1 ng/ml) or IL-1β (10 pg/ml), cytokine alone or in combination, IL-17A+TNF, IL-17F+TNF, IL-17A+IL-1β, IL-17F+IL-1β, IL-17A+TNF+IL-1β or IL-17F+TNF+IL-1β. The production of IL-6 **(A)** and IL-8 **(B)** was measured by ELISA in the supernatants after 48 h. ^*^p ≤ 0.05, ^*^compares with control, ^#^compares conditions. Results are represented as mean ± SEM. *n* = 3. Duplicates were done in each experiment.

IL-17 has an effect on liver inflammation and migration of immune cells (18, 19). IL-17 and TNF have a synergistic effect on hepatocytes (20). In this context, the effects of IL-17A and IL-17F on hepatocytes were compared alone and in combination with TNF or IL-1β. The induced effects were compared to those induced on synoviocytes, same effects were represented by black frame and different effects were represented by filled gray frame in Figure 5.

As for synoviocytes, the four cytokines alone induced an increase of IL-6 production (*p* ≤ 0.03, Figure 5A), with no effect on IL-8 secretion (Figure 5B). The combination of TNF and IL-1β had a synergistic effect on both IL-6 and IL-8 production while it induced only an additive effect in synoviocytes. The combination of TNF and IL-17A also showed a synergistic effect on IL-6 and IL-8 secretion, as for synoviocytes (IL-6, *p* ≤ 0.03, Figure 5A; IL-8, *p* ≤ 0.03, Figure 5B). A similar synergistic effect was observed between IL-17A and IL-1β (IL-6, *p* ≤ 0.03, Figure 5A; IL-8, *p* ≤ 0.03, Figure 5B), while it was an additive effect in synoviocytes. The combination of the three cytokines, IL-17A/TNF/IL-1β increased IL-6 and IL-8 secretion with a synergistic effect compared to cytokine alone.

The combination of IL-17F and TNF had a synergistic effect on IL-6 and IL-8 production, weaker than IL-17A/TNF for IL-6 production but similar to IL-17A/TNF for IL-8 (IL-6, *p* ≤ 0.03, Figure 5A; IL-8, *p* ≤ 0.03, Figure 5B), while in synoviocytes, the IL-17F/TNF synergistic effect was observed only on IL-6 production. Unlike synoviocytes, the combination IL-17F/IL-1β had a synergistic effect, but weaker than IL-17A/IL-1β for both IL-6 and IL-8 secretion (IL-6, *p* ≤ 0.03, Figure 5A; IL-8, Figure 5B). The addition of the three cytokines IL-17F/TNF/IL-1β increased IL-6 while IL-8 production was like the IL-17F/TNF condition.

In conclusion for hepatocytes, the four cytokines alone increased IL-6 but not IL-8 release. As for synoviocytes, IL-17A and TNF had a synergistic effect on IL-6 and IL-8 productions, while unlike synoviocytes the combination IL-17F/TNF had also a synergistic effect on IL-6 and IL-8 productions and not only on IL-6. The combinations IL-17A/IL-1β and IL-17F/IL-1β had a synergistic effect on IL-6 and IL-8, unlike synoviocytes where they induced only an additive effect.

### Cell-Type Dependent Effects

**Figure 6:**
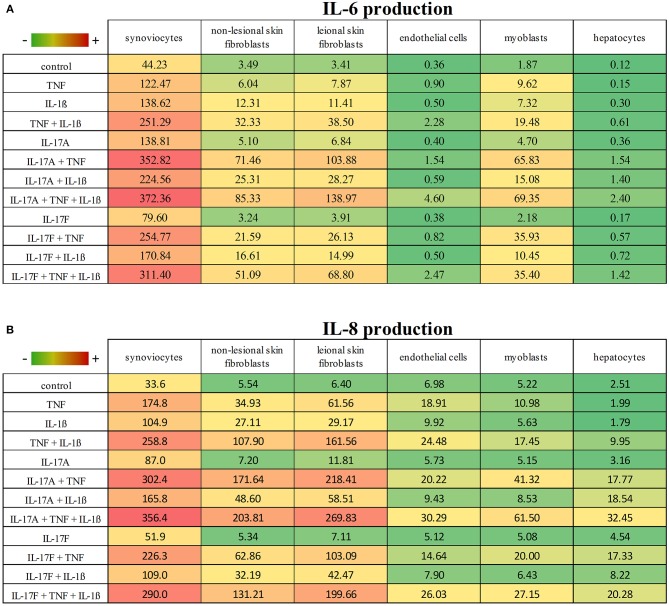
IL-6 and IL-8 production according to cell types and cytokine stimulation. This is a summary of the production of IL-6 **(A)** and IL-8 **(B)** according to cell types and cytokine stimulation. Results are represented as mean, with a color gradient.

**Figure 7:**
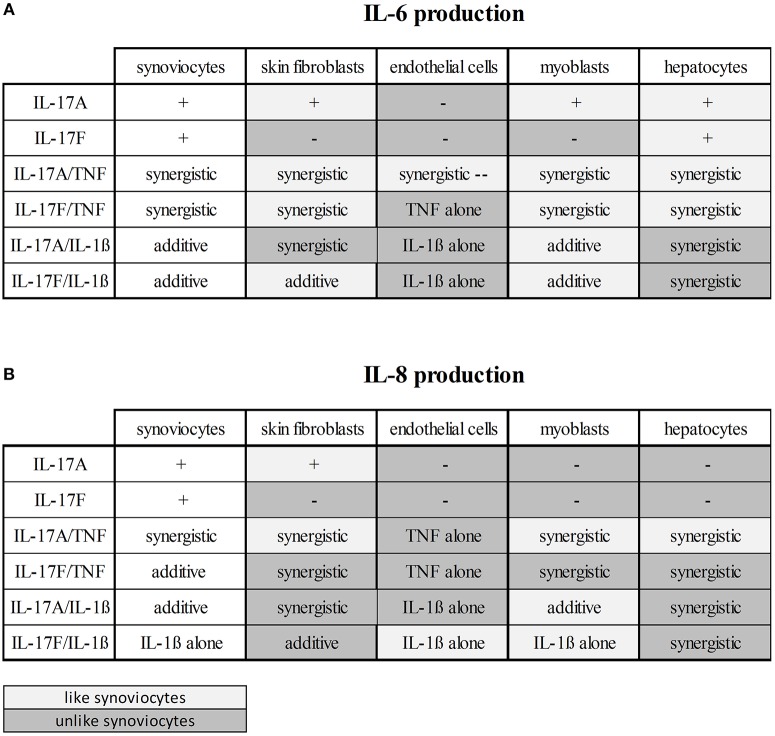
Cytokine stimulation effects according to cell types. This is a summary of the effects on IL-6 **(A)** and IL-8 **(B)** production of the cytokine stimulation depending on cell types. The effects on synoviocytes were taken as reference. The boxes in light gray represent the similar effects and the boxes in dark gray represent the different effects, compared to synoviocytes.

These results taken together showed that cytokine stimulation effects were dependent on cell types (Figures 6, 7). The level of production of IL-6 and IL-8 was also dependent on cell types (Figure 6). Synoviocytes secreted higher concentrations of both IL-6 and IL-8. Endothelial cells and hepatocytes were the weaker producers. Furthermore, heterogeneity was observed in the specific cytokine production (Figure 7). For IL-6 secretion, cytokine stimulation effects on synoviocytes were rather similar on myoblasts, followed by skin fibroblasts and hepatocytes, while they were different on endothelial cells (Figure 7A). For IL-8, heterogeneity was stronger than for IL-6 (Figure 7B) as the closest response to that of synoviocytes were myoblast responses but with only half in common. Thus, there was a real heterogeneity in cytokine responses depending on cell types but also on the produced cytokine.

## Discussion

The aim of the study was to compare the effect of IL-17A and IL-17F, in combination or not with TNF and/or IL-1β, on different cell types to have a global vision of their effects depending on cytokine interactions and cell type. To reduce variability between experiments, the very same culture conditions were used with the use of the same batch of cytokines, and cytokine levels measured with the same ELISA.

IL-17A and IL-17F, alone, increased significantly the production of IL-6 and IL-8 by synoviocytes. As expected, the effect of IL-17A was stronger than that of IL-17F. Nevertheless, even if the effect of IL-17F is weaker than that of IL-17A, IL-17F local production could be higher because of the higher number of IL-17F-producing cells (8). In other cell types, results were different. In hepatocytes IL-17A and IL-17F induced a significant increase of IL-6 but not IL-8. In both non-lesional and lesional skin fibroblasts, IL-17A but not IL-17F increased significantly IL-6 and IL-8 release. In myoblasts, IL-17A had an effect only on IL-6 secretion. In endothelial cells IL-17A or IL-17F had no effect. Thus, the effect of IL-17A or IL-17F alone depends on cell type (Figures 6, 7).

This could be due to a difference in receptor expression and function, as both cytokines have common transducing pathways through the common receptor composed by IL-17RA and IL-17RC. Indeed, in RA synoviocytes, IL-17RA and IL-17RC are overexpressed (21) and this is consistent with the significant response to IL-17A and IL-17F. Moreover, IL-17RA expression is regulated. For example, IL-15 and IL-21 upregulate IL-17RA expression while phosphoinositide 3 kinase (PI3K) limits it (22, 23). Furthermore, the level of IL-17RA expression is biologically important as the IL-17A-induced response is correlated to the expression level of the receptor. High levels of IL-17RA are required for an efficient IL-17A response (24–26). Furthermore, even if both cytokines bind the same receptor, the weaker response to IL-17F could be explained by receptor affinity. Indeed, although IL-17A and IL-17F bind with an equal high affinity the subunit IL-17RC, IL-17F binds with a lower affinity to the subunit IL-17RA compared to IL-17A (27, 28). Thus, differences in IL-17A or IL-17F responses could result from differences in receptor expression level and receptor affinity.

The next step was the combination of IL-17A and TNF. It had a synergistic effect on IL-6 production in all cell types. These results are in line with previous studies (14, 20, 29–31). This synergistic effect may due to different mechanisms. For example, in synoviocytes, IL-17 induces the up-regulation of the TNF receptor II (TNFR-II) which contributes to the synergistic effect (8). This mechanism is not present in hepatocytes as IL-17A alone does not induce TNFR-II (20). Other mechanisms include increased mRNA stability, notably for IL-6 and IL-8 in different cell types (20, 32–35). These different mechanisms in the IL-17A/TNF synergy could explain the different level of synergistic effect depending on cell type. As for IL-6, IL-17A/TNF induced a synergistic effect on IL-8 production in all cell types, except for endothelial cells (Figure 7). This lack of synergistic effect could be explained by the concentration of TNF (1 ng/ml) which is active enough to induce a high IL-8 release by endothelial cells.

Regarding the IL-17F/TNF combination, it had a synergistic effect on IL-6 and IL-8 production by skin fibroblasts, myoblasts and hepatocytes, and on IL-6 secretion by synoviocytes. For endothelial cells, the clear effect of TNF alone could explain the lack of synergistic effect between IL-17F and TNF on IL-6 and IL-8 secretion by endothelial cells. The level of synergistic effect was stronger between IL-17A and TNF than between IL-17F and TNF, in all cell types (Figures 6, 7).

The effect of the combination IL-17A/IL-1β was more dependent on cell types than that of IL-17A/TNF (Figure 7). A synergistic effect was observed in skin fibroblasts and hepatocytes, but an additive effect for synoviocytes, endothelial cells and myoblasts.

In this study, the focus was put on cytokine production selected as key cytokines. Obviously, the combination of these cytokines can have a much larger effect on other genes. Microarray studies have been performed with synoviocytes and endothelial cells using the same culture conditions (8, 36). In this study, IL-17A and IL-17F have a similar regulatory effect on RA synoviocytes, with a stronger effect of IL-17A. Of 601 genes considered as significantly regulated by IL-17A and/or IL-17F, 70.6% (424 genes) were regulated similarly by IL-17A and IL-17F while 27.4% (165 genes) were specifically regulated by IL-17A and only 1.8% (11 genes) by IL-17F (8). In combination with TNF, IL-17A, and IL-17F induced similar expression profiles. Among the genes synergistically induced by IL-17A or IL-17F and TNF, IL-6 and IL-8 were also identified from the microarray results, which is consistent with our results.

Studies on either cytokine production or gene regulation provide consistent results on IL-17A and IL-17F. Alone, IL-17A and IL-17F had a similar effect, with weaker expression for IL-17F. With TNF, this difference between both cytokines was clearly less important, the combination of IL-17A or IL-17F and TNF inducing similar profiles. This indicates that in any inflammatory context, the contribution of IL-17F may be important, due its higher levels than IL-17A, and to the presence of pro-inflammatory cytokines such as TNF or IL-1β. New results with combined inhibition of IL-17A and IL-17F are in line with these *in vitro* results (2).

In conclusion, IL-17A and IL-17F act in a very similar way on different cell types, by inducing IL-6 and IL-8 pro-inflammatory cytokine production. Even if IL-17A induces a stronger response than IL-17F, this difference is reduced by the presence of synergistic interactions with other cytokines, specifically with TNF. The effect of these cytokine combinations is clearly dependent on the cell types. These differences between cell types may explain the different response to treatment according to the primary cell type involved in each disease.

## Data Availability

The datasets generated for this study are available on request to the corresponding author.

## Ethics Statement

For the different cell types, each individual signed an informed consent form. The protocol was approved by the Ethics Committee of the Hospitals of Lyon for the protection of persons participating in biomedical research under number AC-2016-2729.

## Author Contributions

MN carried out the experiments and drafted the manuscript. AB participated in the experiments. PM conceived the study and helped to draft the manuscript. All authors read and approved the final manuscript.

### Conflict of Interest Statement

The authors declare that the research was conducted in the absence of any commercial or financial relationships that could be construed as a potential conflict of interest.
